# Hand–Object Pose Estimation Based on Anchor Regression from a Single Egocentric Depth Image

**DOI:** 10.3390/s25226881

**Published:** 2025-11-11

**Authors:** Jingang Lin, Dongnian Li, Chengjun Chen, Zhengxu Zhao

**Affiliations:** School of Mechanical and Automotive Engineering, Qingdao University of Technology, Qingdao 266520, China; jinganglin163@163.com (J.L.); chencj@qut.edu.cn (C.C.); zhaozhengxu@qut.edu.cn (Z.Z.)

**Keywords:** hand pose estimation, object pose estimation, convolutional neural network, anchor regression, depth image

## Abstract

To precisely understand the interaction behaviors of humans, a computer vision system needs to accurately acquire the poses of the hand and its manipulated object. Vision-based hand–object pose estimation has become an important research topic. However, it is still a challenging task due to severe occlusion. In this study, a hand–object pose estimation method based on anchor regression is proposed to address this problem. First, a hand–object 3D center detection method was established to extract hand–object foreground images from the original depth images. Second, a method based on anchor regression is proposed to simultaneously estimate the poses of the hand and object in a single framework. A convolutional neural network with ResNet-50 as the backbone was built to predict the position deviations and weights of the uniformly distributed anchor points in the image to the keypoints of the hand and the manipulated object. According to the experimental results on the FPHA-HO dataset, the mean keypoint errors of the hand and object of the proposed method were 11.85 mm and 18.97 mm, respectively. The proposed hand–object pose estimation method can accurately estimate the poses of the hand and the manipulated object based on a single egocentric depth image.

## 1. Introduction

The hand is an important tool that humans use to perceive and interact with the world. To precisely understand the interaction behaviors of humans, a computer vision system needs to accurately acquire the pose information of the hand and the manipulated object. Hand–object pose estimation is one of the most important research topics in the field of computer vision, which can be used for robot learning from demonstration, human–computer interaction and augmented reality. However, hand–object pose estimation based on computer vision is still a challenging task due to complicated hand structures, the changing poses of the hand and object, their mutual occlusion, and so on.

Numerous studies have recently focused on independent hand pose estimation [1,2,3,4,5,6,7] and object pose estimation [8,9,10,11]. Although these methods have achieved outstanding results, they rarely involve hand–object interaction cases. Moreover, these methods usually result in poor pose estimation when there is mutual occlusion between the object and hand [12]. Methods that estimate hand and object poses simultaneously fulfill the needs of real scenes better. Sridhar et al. [13] demonstrated that a method that considers hand–object interactions can more accurately predict poses than a pose estimation method that ignores hand–object interactions.

A few studies have investigated hand–object pose estimation tasks and focused on the occlusion problem. Some methods [14,15,16] have adopted multi-camera systems to decrease the influence of occlusions on pose estimation by using images from different viewpoints. However, these methods require the synchronous calibration of multiple cameras, which is expensive and complicated. Some methods are also based on tracking [17,18]. The performance of these methods can be determined by tracking algorithms using the time constraint on the continuous frames of an input image sequence. Nevertheless, methods based on tracking usually require initialization or user-provided prior poses. Additionally, they struggle with the accumulation of tracking errors and automatic recovery after tracking failures. If the poses of the hand and the manipulated object can be estimated directly from a single frame of an image sequence, it is not essential for these methods to use previous frames or prior poses, making them more robust. Due to the complexity of the task, deep learning is the only way to achieve this [19].

As convolutional neural networks (CNNs) are applied extensively, some studies have utilized CNNs to resolve the hand–object pose estimation task. Xiong et al. [20] proposed a hand pose estimation method based on anchor regression. They used anchor points that were uniformly distributed on a depth image to regress the locations of hand joints. However, this method ignored the manipulated object and only estimated the pose of the hand. Oberweger et al. [21] proposed a hand–object pose estimation method based on multiple CNNs, designing a feedback loop to continuously optimize the initial estimation results. However, this method involves several module networks that are trained independently, thus suffering from the problem of complicated training. Moreover, its pose predictors estimate the hand and object independently without considering their mutual relationship. As a result, the pose predictors have poor prediction accuracy, and the results need to be optimized by a feedback loop. The H+O [22] method can predict the poses of a hand and its manipulated object based on egocentric RGB images. It improves pose estimation results using a recurrent neural network (RNN) trained with the pose estimation results of successive frames. However, this method requires continuous video frame inputs to introduce time constraints to optimize the pose estimation results. It is important to note that the initial estimation results of these methods that simultaneously consider the hand and object are not particularly satisfactory, and they require optimization in the follow-up process.

Hand–object pose estimation from egocentric cameras is crucial for robot learning from human demonstration and augmented reality. However, it is even more challenging as the hand is often occluded by the manipulated object or the viewpoint [22]. In this study, a method based on anchor regression is proposed to estimate the hand and object poses from a single egocentric depth image. This method can improve the generalization ability of the model through ensemble learning. Using anchor regression, the method predicts the 3D locations of keypoints by summarizing the prediction results of multiple anchor points. Specifically, anchor points are uniformly distributed on the input image at a high density. The anchor points at different positions predict the locations of all keypoints of the hand and the manipulated object by extracting different local image features. As the contribution of anchor points is different for predicting different keypoints, the method allocates different weights for each anchor point to different keypoints. Finally, all anchor point outputs are summarized under the guidance of the anchor point weights, and hand and object pose prediction is achieved. Additionally, the images taken by depth cameras usually contain disordered background information. Therefore, a hand–object 3D center detection method based on YOLOv3 [23] is proposed to avoid the influence of irrelevant image information on the accuracy of pose estimation. By calculating the hand–object center from the images of interactions, local images only containing the hand and object can be quickly extracted from the original depth images. Thus, the hand–object pose estimation framework in this study consists of a 3D center detection method and a hand–object pose estimation method based on anchor regression.

The major contributions of this study are as follows:

- A hand–object pose estimation method based on anchor regression is proposed. The pose of the manipulated object is parameterized with the same number of keypoints as the hand counterpart, and the keypoint locations of the hand and object are estimated simultaneously in the same framework.
- An anchor point weight allocation scheme is constructed considering the anchor point locations and keypoint prediction errors of both the hand and object.
- A 3D center detection method based on YOLOv3 is proposed that can estimate the 3D location of the hand–object center efficiently. With this method, local images only containing the hand and object can be quickly extracted from the original depth images to avoid the influence of irrelevant image information on the accuracy of pose estimation.
- The proposed hand–object pose estimation framework can simultaneously estimate the poses of the hand and the manipulated object from a single-frame egocentric depth image. It can also provide high prediction accuracy without introducing additional time constraints.

## 2. Related Work

### 2.1. Hand Center Detection

In real scenes, the original images captured by depth cameras usually contain irrelevant background information (other objects or the environment). Therefore, it usually has to detect the location of the hand in the original image for background removal before pose estimation. Oberweger et al. [24] proposed a method for calculating the 3D hand centroid. To crop the hand, they extracted a 3D cube that used the centroid of the hand as the center from the depth image. However, this method assumes that the hand is the closest object to the camera. Therefore, the minimum depth in the depth image is usually set as the initial position of the iteration process. Nevertheless, the locations of the hand and the manipulated object are changeable in real scenes. The minimum depth may not always be near the hand, and it is difficult to calculate the centroid of a hand accurately if this minimum depth is used as the initial position of the iteration algorithm. Zhou [25] proposed an iterative cropping algorithm (ICA) for hand cropping. The ICA uses the rough hand cropping box of the HANDS2019 [26] dataset as the algorithm input, presetting the size of the sampled hand region and the depth threshold to judge the termination of iteration. First, it acquires a sampled centroid through the sampled hand region. Second, the depth of the sampled centroid is compared with the depth threshold. If the threshold requirement is not met, the sampling size is reduced continuously until the iteration termination condition is satisfied. In order to ensure that the cropped image only contains the hand, the ICA adjusts the location of the hand centroid and the size of the 3D cropping box continuously through repetitive iterations. However, it is impossible to acquire a rough cropping box that contains the location information of the hand in real scenes. Moreover, the above methods only locate the centroid of the human hand and are ineffective under the hand–object interaction state. In recent years, CNN architectures such as YOLO variants have achieved significant results in real-time visual analysis in multiple fields [27]. To address the aforementioned issues, a hand–object 3D center estimation module based on YOLOv3 is proposed in this study. It can detect the hand and the manipulated object directly from egocentric depth images and estimate their depth centers for cropping.

### 2.2. Hand Pose Estimation

3D hand pose estimation is an important research problem in the field of computer vision. The research findings have been extensively used in various fields, such as human–machine interaction and robot imitation learning. Recently, deep learning methods, especially CNNs, have been widely applied to the task of 3D hand pose estimation. Methods based on CNNs can be divided into two types according to the network outputs: those based on detection and those based on regression.

Methods based on regression [4,28,29,30,31] establish direct mapping from the input images to the hand poses by CNNs. The hand pose parameters or the 3D coordinates of hand joints are regressed based on a fully connected layer (FCL). These methods can effectively extract the global features of hand joints and demonstrate better performance when the hand is seriously occluded. However, the FCL may destroy the spatial structure of the feature map when aggregating the global features, making it difficult to learn the highly nonlinear mapping from the image feature space to the hand joint space. Using initial hand pose estimation results for guidance, Chen et al. [28] extracted the effective area of the feature map output by the CNN. They proposed a hierarchical fusion method for the effective feature areas. First, this method integrates the joint features on the same finger using the FCL. Then, it integrates the features of different fingers, and the hand poses are regressed by the integrated features. Hand PointNet [4] uses a point cloud that is transformed by the depth images as the input and extracts the hand features using a hierarchical PointNet [32]. Finally, the hand joint positions are directly regressed through the FCL. Madadi et al. [30] constructed a CNN with a tree-like hierarchical structure, where the different branches in the network are responsible for regressing the local poses of different fingers. The features from all branches are integrated by the FCL to estimate hand poses. Cheng et al. [29] regressed the 3D positions of hand joints from a normalized 3D hand point cloud. They proposed a decoder based on folding to fold 2D hand skeletons to the 3D positions of corresponding joints.

Methods based on detection [33,34,35] usually produce dense estimation results and then deduce the joint coordinates through post-processing. Li et al. [33] used 3D hand point cloud data as the network input. Each point generated a hand pose prediction and the corresponding weight estimation of pose parameters. They finally calculated the weighted sum of the hand pose prediction of all points and obtained the final prediction result. Based on the re-parameterization of hand poses, SRN [35] stacks multiple regression networks. This method can obtain 3D heat maps and unit vector fields of hand joint positions through repetitive regression, providing more accurate pose estimation results. Huang et al. [36] proposed an adaptive weighted regression (AWR) method that predicted the positions of hand joints and estimated the corresponding weights for each pixel of the input image. The final prediction results were gained through the weighted sum of the pose estimations of all pixels.

### 2.3. 6D Object Pose Estimation

In recent years, CNNs have been extensively used in the field of 6D object pose estimation. PoseCNN [9] estimates the 6D pose of a target object through RGB images. First, the planar position of the target center was acquired through semantic segmentation. Second, the depth of the target center was estimated according to the segmentation result to obtain the 3D position of the target center. Finally, a regression on the rotation matrix in the quaternion form was carried out using the semantic segmentation result, the target center, and the features extracted by the network. SSD-6D [37] extended the 2D object detector SSD [38] to solve the 6D object pose estimation task. The 3D rotation pose estimation process was transformed into a classification problem by discretizing the rotating space. BB8 [39] segments objects from images using CNNs and estimates the projecting positions of the 3D bounding box corners of the target objects on the 2D images. It then obtains the 6D poses of the target objects using the PnP algorithm. PVNet [40] is a method based on keypoints that predicts the vectors pointing to keypoints for each pixel of the target object through CNNs. Keypoint locations are obtained through voting based on the RANSAC [41] algorithm. Finally, the poses of the objects are resolved using the PnP algorithm. Song et al. [42] proposed a method for object pose estimation based on a hybrid intermediate representation. They regressed object poses through the keypoints, edge vectors, and symmetric relation features.

### 2.4. Hand–Object Pose Estimation

Mutual occlusion happens frequently between hands and manipulated objects during their interactions. Therefore, hand–object pose estimation is still a challenging task in the field of computer vision. Hasson et al. [43] proposed a contact loss based on the physical constraint that hand–object contacts only occur on the surface of the object to restrict the network to generating reasonable pose estimations. The H+O method [22] proposed a CNN that simultaneously estimated the locations of the hand joints and the keypoints of the object’s bounding box. Moreover, this method trained an RNN model through pose estimation results to recognize types of actions. It also introduced time constraints to further optimize the hand–object pose estimation results. To acquire higher accuracy, Oberweger et al. [21] built a feedback loop based on CNNs to optimize the initial results of the hand and object pose predictors. This feedback loop is primarily composed of three parts: predictors, synthesizers, and updaters. First, the hand and object poses are estimated by the predictors from the input depth images. Then, the synthesizers synthesize the corresponding depth images according to the poses output by the predictors. Using the synthesized depth images and the input depth images as the inputs, the updaters provide updates to the pose estimation results. The feedback loop adjusts the results of the predictors according to the updates and imports them into the synthesizers, repeating the above steps several times. Hope-Net [44] reported that the graph-based method can naturally model the skeleton and kinematic constraints between joints, proposing a hand–object pose estimation method based on graph convolution networks (GCNs). This method is composed of two adaptive GCNs. One estimates the 2D coordinates of the hand keypoints and the bounding box corners of the object. The other transforms the 2D coordinates into 3D coordinates. In recent years, a lot of progress has been made in the field of 3D hand–object pose estimation and the corresponding datasets [45,46,47,48,49,50]. However, most of the above methods rely on RGB or RGB-D inputs and often integrate temporal information or multiple camera views, which differ from the method proposed in this paper. This paper focuses on the 3D hand–object pose estimation from single-frame egocentric depth images.

## 3. Method

The proposed method can detect the location of the hand–object center and simultaneously estimate the poses of the hand and the manipulated object based on egocentric depth images. In this section, the overall framework of the proposed method is introduced, followed by a detailed description of the two components of the proposed method: the hand–object 3D center detection module and the hand–object pose estimation module based on anchor regression.

### 3.1. Overall Framework

**Figure 1:**
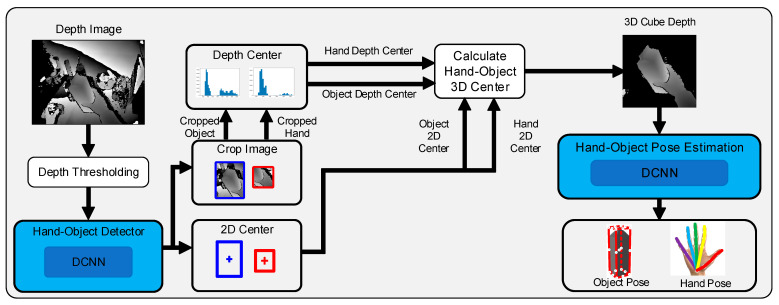
Flowchart of the hand–object pose estimation method based on anchor regression.

The flowchart of the proposed hand–object pose estimation method is shown in Figure 1. First, the original depth images were used as the initial inputs, and part of the disordered background in the images was removed using the simple depth threshold method. The hand–object center detection module received the processed depth image as the input and calculated the hand–object 3D center. Then, the original depth image was cropped by the hand–object center using the 3D cropping method, obtaining a cropped depth image that only contains the hand and the manipulated object. Finally, the hand–object pose estimation module based on anchor regression received the cropped depth image as the input to estimate the poses of the hand and object.

The proposed method expressed the poses of the hand and the manipulated object in a 3D keypoint form. Two groups of keypoints (N1 and N2) were used to express the poses of the hand and object, respectively. The hand–object pose estimation module based on anchor regression places anchor points on the cropped depth image at a fixed pixel interval. Due to different locations on depth images, anchor points may generate different position predictions and weight predictions for each keypoint. The hand–object pose estimation module aggregates anchor point outputs through a weighted sum and calculates the locations of 3D keypoints of the hand and the manipulated object.

### 3.2. Hand–Object 3D Center Detection Module

For the hand–object pose estimation task, segmenting the hand and the manipulated object accurately from the original image is vital for the subsequent pose estimation. The original images of real hand–object interactions captured by the depth camera usually contain disordered backgrounds. Therefore, the hand and the manipulated object have to be segmented from the original depth image before pose estimation, which can avoid the influence of disordered background information on pose estimation. This improves the accuracy and generalization of the model. Based on the accurate 3D target center, the corresponding target image can be cropped from the original depth image. Therefore, a 3D center detection method is proposed to detect the medium location of the centers of the hand and object, which is used as the 3D center to simultaneously segment the hand and object from the original depth image. The proposed hand–object 3D center detection method is divided into three parts: 2D center estimation, depth center estimation, and 3D center calculation. This method can estimate the hand–object 3D center based on the input depth image and segment the hand and manipulated object from the depth image by using the 3D center.

#### 3.2.1. Hand–Object 2D Center Estimation

To segment the hand and the manipulated object from the original depth image, accurate estimations of their positions are essential. The 2D centers of the hand and object were estimated based on the YOLOv3 detection algorithm. Thus, the 2D bounding boxes of the hand and object can be acquired, and their 2D centers in the image coordinate system can be calculated. The original depth images usually contain complicated background information, and irrelevant objects in the far background may influence the detection algorithm. As the irrelevant background and the hand are typically not at the same depth intervals, a depth threshold was established to separate the disordered background from the original depth image. The acquired foreground depth images were then used as the inputs of the YOLOv3 model. In this study, the bounding boxes of the hand and object predicted by YOLOv3, as well as the corresponding 2D centers, were used as the outputs of this module.

Based on CNNs, the YOLOv3 uses Darknet-53 as the backbone network for feature extraction. Darknet-53 with residual structures can improve feature extraction abilities and prevent the gradient vanishing and gradient explosion with the deepening of the network. To detect different-scale targets effectively, YOLOv3 integrates different scales of feature maps by using the feature pyramid network (FPN) structure for feature extraction. In addition, YOLOv3 outputs 3 scales of predicted feature maps at the network output step for different target scales.

#### 3.2.2. Hand–Object Depth Center Estimation

**Figure 2:**
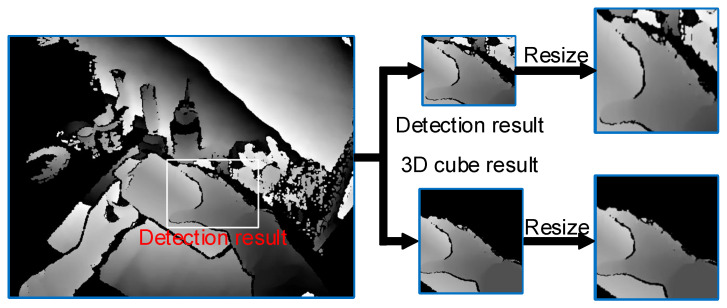
Hand images cropped from the original depth image using different methods.

After detecting the positions of the hand and the manipulated object, it is necessary to crop the images to a fixed size from the original depth images for the follow-up pose estimation. An obvious way to accomplish this is to directly use the detected 2D bounding boxes and 2D centers of the hand and manipulated object for cropping: the image region containing the hand and object is cropped and then adjusted to the appropriate size. However, this cropping method has two shortcomings. First, the hand and object have varied poses, resulting in uncertain sizes for the detected 2D bounding boxes. As shown in Figure 2, adjusting the sizes of the cropped images using the 2D bounding boxes may cause irregular distortion and deformation in the hand and object. Moreover, directly cropping the original depth images in 2D may retain deep background information, which could introduce irrelevant noise to the pose estimation.

To address these problems, the 3D cropping method was applied in this paper. A 3D cube was extracted at the 3D location of the hand–object center, which cropped a background-free and fixed-size image from the original depth image. The key to extracting an appropriate 3D cube is acquiring the 3D location of the hand–object center. The 2D centers of the hand and object $\left(x_{center},y_{center}\right)$ can be acquired by the hand detector and the object detector. It usually leads to significant errors if the depth values of the 2D centers are used as the depth values ($z_{center}$) of the 3D centers. This is due to (1) inconsistencies, such as missing data and noise in the original depth images, and (2) deviation between the depth values of the 2D centers and the actual 3D centers. Hence, the hand–object depth center estimation module is proposed to estimate the depth values of the hand center and object center.

**Figure 3:**
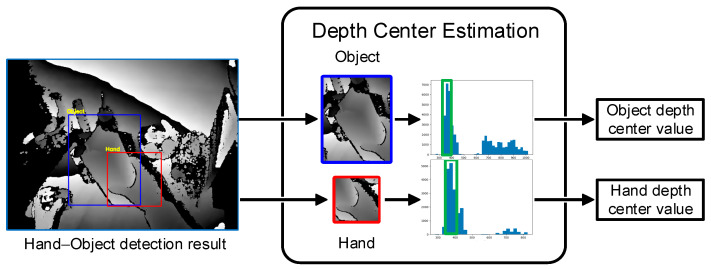
Flowchart of hand–object depth center estimation.

A flowchart of hand–object depth center estimation is shown in Figure 3. First, the original depth images were cropped according to the detected 2D bounding boxes of the hand and object to obtain a hand-cropped image and an object-cropped image. Second, two depth histograms were established to calculate the number of pixels in each depth interval of the two images. Finally, the depth center values of the hand and object were calculated according to the medians of the 3 depth intervals with the most pixels in each histogram and the corresponding numbers of pixels. The depth center values were calculated according to the following equation:

(1) $$Center_{d}=\frac{\left(D_{I1}N_{I1}+D_{I2}N_{I2}+D_{I3}N_{I3}\right)}{N_{I1}+N_{I2}+N_{I3}}$$

where $Center_{d}$ is the depth value of the estimated depth center. $D_{I1}$, $D_{I2}$, and $D_{I3}$ are the medians of the 3 depth intervals with the most pixels. $N_{I1}$, $N_{I2}$, and $N_{I3}$ are the numbers of pixels in the 3 depth intervals.

#### 3.2.3. Hand–Object 3D Center Calculation

Using the 3D centers of the hand and the manipulated object as inputs, the hand–object 3D center was calculated according to the following equation:

(2) $$Center=\frac{Center_{H}+Center_{O}}{2}+D_{b}$$

where Center, $Center_{H}$, and $Center_{O}$ are the hand–object 3D center, the hand 3D center, and the object 3D center, respectively. $D_{b}$ is the depth offset to adjust the position for the hand–object 3D center.

The 3D cropping range for the depth image was calculated using the hand–object 3D center and a 3D cube with a fixed size. The 3D cropping range covered two parts: the plane cropping range and the depth cropping range. Cropped images with a fixed size were acquired from the original depth images according to the plane range. A depth threshold was established according to the depth range to separate invalid background information from the images. The plane cropping range and depth cropping range were calculated using the hand–object 3D center and 3D cube according to the following equations:

(3) $$\begin{array}{c} \begin{array}{c} u_{start}=Center_{x}-\frac{Cube_{x}}{2}\times \frac{f_{x}}{Center_{z}}\\ u_{end}=Center_{x}+\frac{Cube_{x}}{2}\times \frac{f_{x}}{Center_{z}}\\ v_{start}=Center_{y}-\frac{Cube_{y}}{2}\times \frac{f_{y}}{Center_{z}} \end{array}\\ \begin{array}{c} v_{end}=Center_{y}+\frac{Cube_{y}}{2}\times \frac{f_{y}}{Center_{z}}\\ d_{start}=Center_{z}-\frac{Cube_{z}}{2}\\ d_{end}=Center_{z}+\frac{Cube_{z}}{2} \end{array} \end{array}$$

where ${\left(u,v\right)}_{strart,end}$ are the start and end values of the *x*-axis and *y*-axis of the plane cropping range. $d_{strart,end}$ are the start and end values of the depth segmentation range. $Cube_{x,y,z}$ represent the size parameters of the 3D cropping cube. $f_{x}$ and $f_{y}$ are the internal parameters of the depth camera.

### 3.3. Hand–Object Pose Estimation Module Based on Anchor Regression

The hand–object pose estimation module based on anchor regression (HOAR) aims to estimate the 3D locations of the keypoints of the hand and manipulated object from egocentric depth images. Unlike anchor-based object detection methods that only predict 2D bounding boxes, in this paper, the anchor regression method predicts 3D offset vectors from uniformly distributed anchor points to keypoints and the corresponding weights. This allows the anchor points to jointly determine the final estimation result through a spatial voting mechanism, thereby obtaining higher robustness in the presence of occlusion and noise. Compared with the single-point regression method, this strategy has better generalization performance for unknown hand–object combinations.

**Figure 4:**
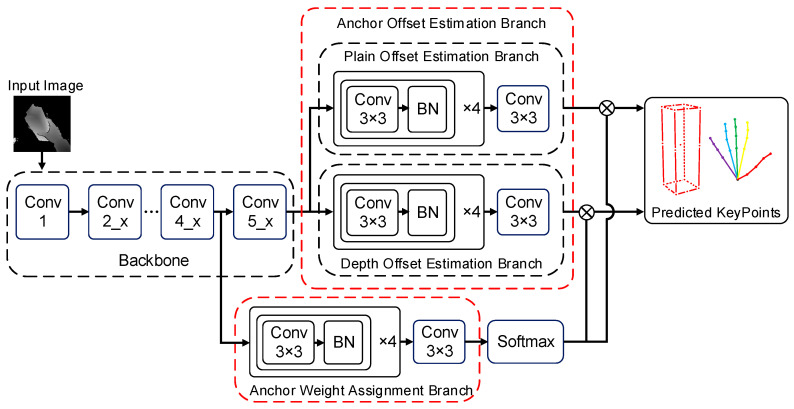
Structure of the HOAR module.

The structure of the HOAR module is shown in Figure 4. This module is composed of a backbone network for feature extraction and two functional branches. The two functional branches include the anchor point offset estimation branch and the anchor point weight estimation branch. The hand–object 3D center and 3D cropping range were acquired from the hand–object 3D center detection module. The cropped images covering the hand and the manipulated object were acquired from the original depth images based on the 3D cropping ranges. Moreover, the cropped images were preprocessed before hand–object pose estimation. First, the cropped images were adjusted to 176 × 176. Second, the depth values of the cropped images were standardized to the range of [−1,1] using the depth value at the hand–object 3D center. For depth images, the above image standardization can help the hand and object maintain constant features when their distances from the camera change, which can accelerate the convergence of the network.

#### 3.3.1. Backbone Network for Feature Extraction

The HOAR module utilized a ResNet-50 [51] pre-trained on ImageNet [52] as the backbone network for feature extraction. ResNet-50 contains many residual structures that can effectively prevent gradient vanishing and gradient explosion induced by the deepening of the network. Moreover, ResNet-50 has a moderate number of parameters and performs stably in pose estimation tasks. It achieves a good balance between feature extraction capability, training stability, and computational efficiency. Meanwhile, its pre-trained weights on ImageNet help accelerate the convergence of the model during the training process. To adapt to the requirements of the hand–object pose estimation task, the backbone network for feature extraction used in the HOAR module is different from the original ResNet-50 network. To retain the spatial information of images and produce 2D feature maps, the FCL of ResNet-50 was removed. Moreover, the convolution step size in the Conv4_x was adjusted to 1. The resolution of the feature map output by the Conv4_x was adjusted from the downsampling of the input depth images by a factor of 32 to downsampling by a factor of 16. The smaller downsampling factor was applied to allow the output feature map to maintain subtler spatial information. Furthermore, the convolution in the Conv4_x layer was replaced with a dilated convolution with a dilation rate of 2 to expand the range of the receptive field of the CNN.

The feature extraction backbone network in the HOAR module has two outputs that are used as the inputs of the anchor point offset estimation branch and the weight estimation branch. Specifically, the output feature map of the Conv4_x layer in ResNet-50 is used as the input for the anchor point weight estimation branch. The branch used this feature map to estimate the weights of each anchor point to different keypoints. Moreover, the output feature map of the Conv5_x layer in ResNet-50 is used as the input for the anchor point offset estimation branch. This feature map was used to estimate the locations of different keypoints for each anchor point.

#### 3.3.2. Anchor Point Offset Estimation Branch

The proposed HOAR module can estimate the keypoints of the hand and the manipulated object according to the dense anchor points of depth images. Each anchor point is equivalent to a local spatial regressor. The 3D locations of different keypoints were predicted by extracting the local context features of anchor points. Finally, the estimation results for the 3D locations of keypoints by all anchor points were acquired through the aggregation method based on the weights of the anchor points. One anchor point was established for every 4 pixels on the depth image. Each anchor point generated the location estimation of different keypoints independently.

**Figure 5:**
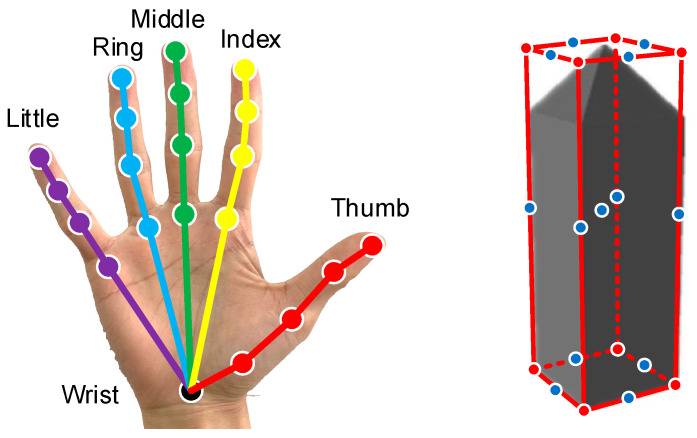
Locations of the keypoints of the hand and the manipulated object.

The anchor point offset estimation branch estimates the 3D offsets between the anchor point and different keypoints. The 3D offsets can be categorized into plane offsets and depth offsets. The estimated plane offsets and depth offsets are affected by different image features. Hence, two parallel branches were established to estimate the plane offsets and depth offsets for each anchor point. The corresponding locations of the hand and the manipulated object for the keypoints to be estimated by the HOAR module are shown in Figure 5. Specifically, the keypoints of the hand correspond to the 3D locations of 21 hand joints, while the keypoints of the manipulated object correspond to the 8 vertices, the midpoint of each edge, and the centroid of the 3D bounding box. The hand and manipulated object poses were estimated in a uniform network framework, and their keypoints were coherently parameterized. The keypoints of the object after coherent parameterization have the same number of parameters as the keypoints of the hand. It is beneficial to balance network learning for hand pose estimation and object pose estimation.

**Figure 6:**
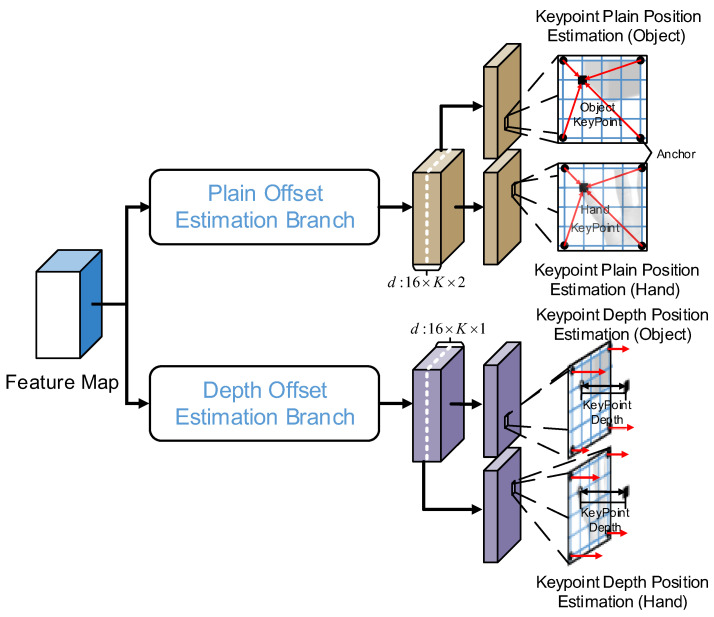
Structure of the anchor point offset estimation branch.

The structure of the anchor point offset estimation branch is shown in Figure 6. This branch uses the output feature map of the Conv5_x layer in the feature extraction backbone network as the input. The plane offset estimation branch and depth offset estimation branch both receive the feature map as an input. The plane offset estimation branch consists of four 3 × 3 convolutions, which are used to further extract the image features. Since the feature map acquired from the backbone network is a downsampling of the input image of the HOAR module by a factor of 16, and the anchor points are established for every 4 pixels, each point on the feature map output by the plane offset estimation branch corresponds to 16 anchor points of different areas. There are K keypoints for both the hand and the manipulated object. The plane offset is expressed by 2 parameters. Therefore, the number of channels produced by the plane offset estimation branch is 16 × K × 2. The structure of the depth offset estimation branch is essentially the same as that of the plane offset estimation branch, except that the depth offset is expressed by 1 parameter. Hence, the number of channels produced by the depth offset estimation branch is 16 × K × 1.

#### 3.3.3. Anchor Point Weight Estimation Branch

Dense estimations for keypoint locations are produced by the anchor points that are uniformly distributed on the depth image. The final prediction results can be acquired through the mean of all anchor point estimations. However, the local image features surrounding the anchor points are different, as each anchor point occupies different spatial locations in the depth image. Moreover, the distance between each anchor point and the keypoints varies. Thus, it is more useful to estimate the location of a keypoint using a closer anchor point. Given this, it is necessary to assign different weights to different keypoints for each anchor point and summarize the estimation results of all anchor points through a weighted sum.

**Figure 7:**
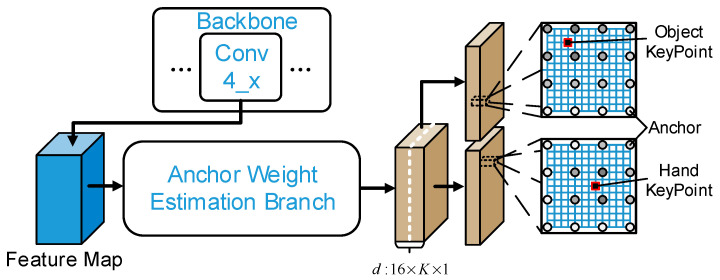
Structure of the anchor point weight estimation branch.

The structure of the anchor point weight estimation branch is shown in Figure 7. This branch assigns different weights to different keypoints for each anchor point. The HOAR module summarizes the keypoint locations estimated by all anchor points based on the weights of anchor points through the weighted sum. This method can significantly reduce the influence of large anchor point prediction errors on the final results. For the weight allocation range, some hand pose estimation methods based on detection restricted the range of weight allocation or set it as a fixed value to reduce calculation expenses and avoid the influence of the detection points with low correlation on estimation results. Nevertheless, these methods may produce poor estimation results when input depth images have noise or missing depth in the weight allocation range. To address this problem, in this paper, we did not restrict the weight allocation range in the anchor point weight estimation branch. The weight allocation range covers all anchor points that are distributed uniformly in the input image. The weights of all anchor points are adjusted automatically through the network learning to produce robust pose estimation results when local images have noise or missing depth. The calculation process for keypoint plane position and keypoint depth position is as follows:

(4) $$\begin{array}{c} \tilde{S}\left(k\right)=\sum_{a\in A}\tilde{W}\left(a,k\right)\left(S\left(a\right)+O\left(a,k\right)\right)\\ \tilde{D}\left(k\right)=\sum_{a\in A}\tilde{W}\left(a,k\right)D\left(a,k\right) \end{array}$$

where A is the set of anchor points. $\tilde{S}\left(k\right)$ is the plane position of keypoint k estimated by all anchor points. $S\left(a\right)$ is the plane position of anchor point a. $O\left(a,k\right)$ denotes the plane offset of keypoint k estimated by anchor point a. $\tilde{D}\left(k\right)$ denotes the depth location of keypoint k estimated by all anchor points. $D\left(a,k\right)$ is the depth offset of keypoint k estimated by anchor point a. $\tilde{W}\left(a,k\right)$ is the normalized weight of keypoint k acquired by anchor point a. The weight normalization of the anchor points was performed via the SoftMax function, as follows:

(5) $$\tilde{W}\left(a,k\right)=\frac{e^{W\left(a,k\right)}}{\sum_{a\in A}e^{W\left(a,k\right)}}$$

where $W\left(a,k\right)$ is the weight of keypoint k estimated by anchor point a.

#### 3.3.4. Loss Function

The loss function of the proposed HOAR module is composed of two parts: the 3D location estimation loss of keypoints and the weight allocation loss of anchor points. The former refers to the error between the 3D locations of keypoints estimated by anchor points and the ground-truth 3D locations of corresponding keypoints, which is used to supervise the anchor point to generate accurate offset estimations. The latter constrains the weight allocation process of the anchor points. It is expected that the network will allocate high weights to anchor points with strong correlations to the keypoints. To better estimate the hand and object poses simultaneously, the pose of the manipulated object was expressed by the same number of keypoints as the pose of the hand. Using the same number of object keypoints as hand keypoints is beneficial for the loss function in balancing network learning between hand pose estimation and object pose estimation.

The 3D location estimation loss of the keypoints ($Loss_{1}$) comprises two parts: the 3D location estimation loss of the hand keypoints ($loss_{H}$) and the 3D location estimation loss of the object keypoints ($loss_{O}$). The calculation formulas of these three losses are as follows:

(6) $$\begin{array}{c} Loss_{1}=\lambda_{O}loss_{O}+\lambda_{H}loss_{H}\\ loss_{O}=\alpha \sum_{k_{o}\in K_{O}}L_{\tau_{1}}\left(\tilde{S}\left(k_{o}\right)-T_{k_{o}}^{v}\right)+\sum_{k_{o}\in K_{O}}L_{\tau_{2}}\left(\tilde{D}\left(k_{o}\right)-T_{k_{o}}^{d}\right)\\ loss_{H}=\alpha \sum_{k_{h}\in K_{H}}L_{\tau_{1}}\left(\tilde{S}\left(k_{h}\right)-T_{k_{h}}^{v}\right)+\sum_{k_{h}\in K_{H}}L_{\tau_{2}}\left(\tilde{D}\left(k_{h}\right)-T_{k_{h}}^{d}\right) \end{array}$$

where $\lambda_{O}$ and $\lambda_{H}$ are the factors balancing the 3D location estimation task of the object keypoints and the 3D location estimation task of the hand keypoints, which were fixed at 1. α is the factor balancing the plane position estimation and the depth position estimation, which was fixed at 0.5. $T_{k_{o}}^{v}$ is the ground-truth plane location of object keypoint $k_{o}$. $T_{k_{o}}^{d}$ is the ground-truth depth location of object keypoint $k_{o}$. $T_{k_{h}}^{v}$ is the ground-truth plane location of hand keypoint $k_{h}$. $T_{k_{h}}^{d}$ is the ground-truth depth location of hand keypoint $k_{h}$. $\tilde{S}\left(k_{o}\right)$ is the predicted plane location of object keypoint $k_{o}$. $\tilde{D}\left(k_{o}\right)$ is the predicted depth location of object keypoint $k_{o}$. $\tilde{S}\left(k_{h}\right)$ is the predicted plane location of hand keypoint $k_{h}$. $\tilde{D}\left(k_{h}\right)$ is the predicted depth location of hand keypoint $k_{h}$. $L_{\tau }$ is the smoothL1 loss function with an adjusting parameter τ, which is calculated as

(7) $$L_{\tau }\left(x\right)=\left\{\begin{array}{cc} \frac{x^{2}}{2\tau }, & \left|x\right|<\tau \\ \left|x\right|-\frac{\tau }{2}, & \left|x\right|\geq \tau \end{array}\right.$$

The adjusting parameter $\tau_{1}$ was fixed as 1 and $\tau_{2}$ was fixed at 3. This is because the keypoints’ changes in depth are more abrupt than their changes in plane.

The anchor point weight allocation loss supervises the network in the process of anchor point weight allocation. The proposed method did not restrict the range of the anchor points for keypoint prediction to improve robustness. However, the anchor point close to the keypoint can provide better offset prediction. Hence, the anchor points close to and with strong correlations to the keypoints can be allocated with relatively high weights. The calculation formula for the anchor point weight allocation loss ($Loss_{2}$) is as follows:

(8) $$Loss_{2}=\lambda_{O}\sum_{k_{o}\in K_{O}}L_{\tau_{1}}\left(\sum_{a\in A}\tilde{W}\left(a,k_{o}\right)S\left(a\right)-T_{k_{o}}^{v}\right)+\lambda_{H}\sum_{k_{h}\in K_{H}}L_{\tau_{1}}\left(\sum_{a\in A}\tilde{W}\left(a,k_{h}\right)S\left(a\right)-T_{k_{h}}^{v}\right)$$

The training of the HOAR module is simultaneously supervised by $Loss_{1}$ and $Loss_{2}$. The total loss function of the module is defined as follows:

(9) $$Loss=\beta Loss_{1}+Loss_{2}$$

where Loss is the total loss of the HOAR module. β is the weight factor balancing the 3D location estimation loss and the anchor point weight allocation loss. This value was fixed at 3.

## 4. Experiments and Discussion

### 4.1. Dataset

To evaluate the proposed hand–object pose estimation method based on anchor regression, a comparison experiment was carried out on the First-Person Hand Action (FPHA) [53] dataset. The FPHA is an open egocentric hand–object interaction dataset, which comprises 1175 RGB-D videos. The FPHA provides accurate labels for 3D hand joint locations and the corresponding interactions for all video frames. Furthermore, FPHA offers the 3D translation and rotation parameters of the manipulated objects in certain video frames for the 6D pose labeling of these objects. Here, the FPHA subset providing the 6D pose labels for the manipulated objects is called FPHA-HO. The proposed method simultaneously estimated the poses of the hand and the manipulated object. Therefore, all follow-up experiments were carried out on the FPHA-HO dataset.

The FPHA-HO dataset contains 21,501 RGB-D images, covering 10 types of interactive actions and four manipulated objects and providing corresponding 3D mesh models of these objects. To make object pose annotation applicable to the method proposed in this study, the 3D mesh model was translated and rotated to the annotated pose, and then, its compact 3D bounding box was calculated. The keypoints of the bounding box were used as the object pose label. This paper follows the FPHA-HO dataset partitioning criteria of the H+O [22] method, with 11,019 frames used for training and 10,482 frames used for testing.

### 4.2. Evaluation Metrics

The following evaluation metrics were applied to evaluate the model’s performance. The mean keypoint error (MKE) is used to express the mean Euclidean distance (unit: mm) between the predicted 3D coordinates and the ground-truth 3D coordinates of all keypoints of the hand and manipulated object. The following is the calculation formula for the MKE:

(10) $$MKE=\frac{1}{K}\times \frac{1}{F}\sum_{k}^{K}\sum_{f}^{F}\sqrt{{\left(\Phi_{fk}^{est}-\Phi_{fk}^{gt}\right)}^{2}}$$

where K is the total number of keypoints. F is the total number of image frames in the test set. $\Phi_{fk}^{est}$ is the estimated 3D coordinate of keypoint k in frame *f*. $\Phi_{fk}^{gt}$ is the ground-truth 3D coordinate of keypoint k in frame *f*.

The percentage of correct keypoint estimation (PCK) is another metric for evaluation. If the error between the predicted keypoint coordinate and the ground-truth keypoint coordinate is smaller than the preset threshold, the 3D location estimation of this keypoint is regarded as correct. The calculation formula of the PCK is as follows:

(11) $$\begin{array}{c} PCK=\frac{1}{K}\times \frac{1}{F}\sum_{f}^{F}\sum_{k}^{K}\delta \left(L_{f}^{k},\theta \right)\times 100\%\\ \delta \left(L_{f}^{k},\theta \right)=\left\{\begin{array}{ccc} 1 & , & L_{f}^{k}\leq \theta \\ 0 & , & L_{f}^{k}>\theta \end{array}\right.\\ L_{f}^{k}=\sqrt{{\left(\Phi_{fk}^{est}-\Phi_{fk}^{gt}\right)}^{2}} \end{array}$$

where θ is the threshold. F is the total number of image frames in the test set. $L_{f}^{k}$ is the absolute distance between the estimated keypoint $\Phi_{fk}^{est}$ and the ground-truth keypoint $\Phi_{fk}^{gt}$.

### 4.3. Experimental Platform and Parameter Settings

The hand–object 3D center detection module received the 640 × 480 depth images as inputs, and the depth threshold for background segmentation was set to 650 mm. This depth threshold was empirically chosen based on the depth distribution of the hand–object interaction areas in the egocentric FPHA-HO dataset, which can effectively distinguish between foreground and background. The 2D hand–object center estimation module was first pre-trained on the Hand Dataset [54], and the pre-learned weights of the Darknet [23] backbone network were used as the initial weights of the follow-up training. The 2D hand–object center estimation module used the stochastic gradient descent (SGD) algorithm as the optimizer. The size of the 3D cropping cube was 350 mm × 350 mm × 350 mm.

The HOAR module adjusted the images cropped by the 3D cropping cube to a fixed resolution of 176 × 176. Meanwhile, the depth of the input images was standardized to [−1,1] according to the center location of the 3D cropping cube. Data enhancement measures were applied to the input depth images. The input images were randomly rotated in a range of [−45°,45°] and randomly scaled in a range of [0.5,1]. Furthermore, Gaussian noise was randomly added to the images at a probability of 0.5. The hand–object pose estimation module was trained using the Adam optimizer. The learning rate was set as 0.0007, and the weight decay was set as 10E−4. The model was trained for 200 epochs on the FPHA-HO dataset. The learning rate decayed to 0.5 times the previous level every 20 epochs.

The proposed modules were all constructed on the Ubantu18.04 system based on the Pytorch deep learning framework. The hardware system was a server equipped with one NVIDIA TITAN GPU, one Intel Xeon E5-2630v4@2.2GHz CPU, and 32 G of memory.

### 4.4. Experimental Results and Analysis

Experiments were carried out on the FPHA-HO dataset. To verify the effectiveness of the proposed hand–object pose estimation method based on anchor regression, it was compared with the state-of-the-art hand–object pose estimation method H+O [22]. To determine the influence of hand–object 3D center location calculation on the hand–object pose estimation results of the proposed method, the ground-truth 3D centers of the hand and manipulated object were used as their ideal centers. The performance of the proposed pose estimation method was compared using the ideal centers and the predicted centers of the hand and object, respectively.

**Figure 8:**
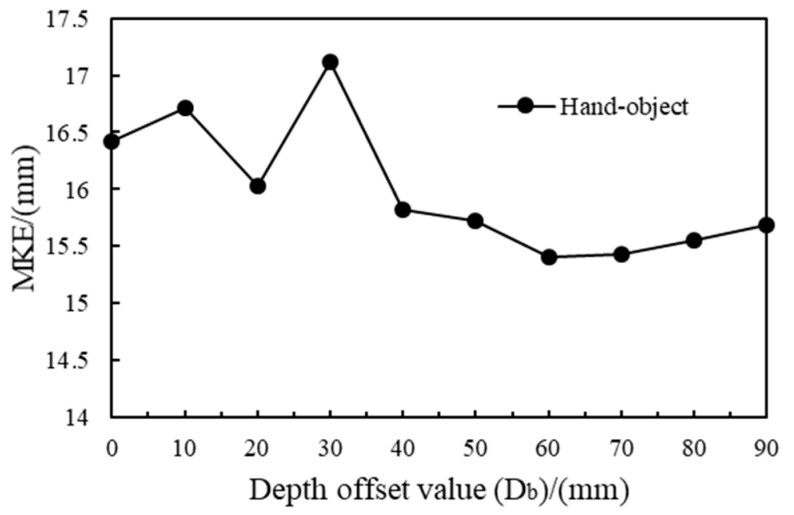
Hand–object MKE with different depth offset values.

The cropped depth output of the hand–object 3D center detection module was adjusted by the depth offset ($D_{b}$). Different $D_{b}$ values would influence the input depth images of the follow-up pose estimation module. Thus, the prediction results of the model on the test set using different $D_{b}$ values are shown in Figure 8. The model achieved the best performance on the test set when $D_{b}$ was 60 mm. Under this condition, the MKE of the hand was 11.853 mm, the MKE of the manipulated object was 18.971 mm, and the hand–object MKE was (11.853 + 18.971)/2 mm = 15.412 mm. Follow-up experiments were carried out under this parameter.

The MKE results of our method and H+O on the FPHA-HO dataset are presented in Table 1. H+O (hand only) and H+O (object only) refer to the model only estimating the hand pose and the model only estimating the object pose in [22], respectively. H+O (base) and H+O (interact) refer to the hand–object pose estimation basic model and the hand–object pose estimation model with temporal reasoning and interaction modeling in [22], respectively. H+O (interact) constructed an RNN and used continuous video frames to learn the interaction between the hand and the manipulated object to further optimize the pose estimation results. Ours (predicted center) denotes the proposed hand–object pose estimation method based on anchor regression using the predicted hand–object 3D center, while ours (ideal center) denotes the counterpart using the ideal hand–object 3D center

As shown in Table 1, the hand pose estimation accuracy and the object pose estimation accuracy of the proposed method on the FPHA-HO test set were better than those of the H+O method. By introducing temporal reasoning and interaction modeling, H+O reduced the influence of hand–object mutual occlusion on pose estimation results based on the time relations of the continuous frames in the videos, resulting in improved pose estimation accuracy. It should be noted that the H+O method estimates poses from an RGB image sequence, while ours uses a single depth image as input. Given the differences in input modalities, the performance comparison in Table 1 should primarily be viewed as a comparative analysis under common task objectives, rather than a same-modal comparison. This comparison aims to demonstrate that the performance of our method can achieve competitive pose accuracy using only a single depth image, without requiring RGB data or temporal information.

**Figure 9:**
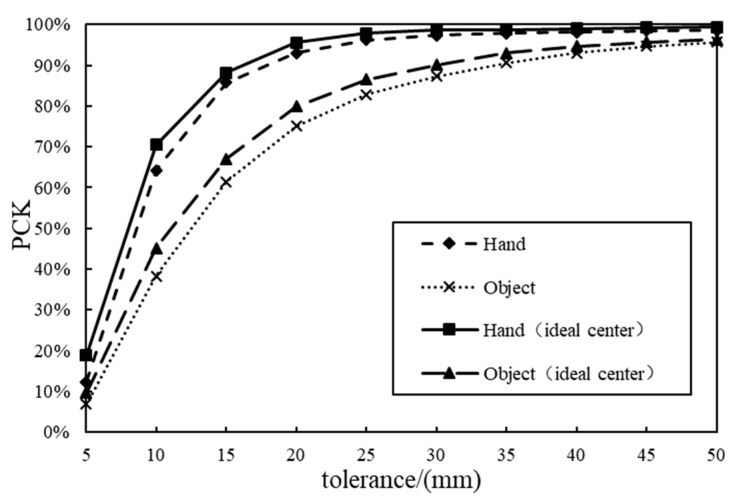
PCK values of the models using different hand–object 3D centers.

When using the ideal hand–object 3D centers, the hand pose and object pose estimation errors were 2.43 mm and 4.35 mm less than those when using the predicted centers. As shown in Figure 9, the PCK of the hand–object pose estimation method using the ideal hand–object 3D centers for cropping was superior to that of the method using the predicted 3D centers under all thresholds. This proves that the pose estimation accuracy could be improved with a more reasonable hand–object 3D center. Compared with ideal hand–object 3D centers, the predicted centers may decrease the pose estimation accuracy of the proposed method. However, it is difficult to acquire ideal centers in real scenes. Nonetheless, the proposed method using the predicted hand–object 3D centers was still superior to the H+O method in terms of pose estimation accuracy.

**Figure 10:**
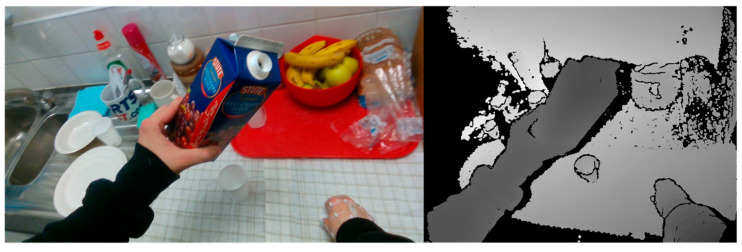
Long distance between the manipulating hand and the object.

Although the method proposed in this paper performed well in most cases, there were still certain specific cases where the performance of 3D hand–object pose estimation was poor. For example, when the hands are severely occluded by large objects or there are missing areas in the depth image, the anchor votes may become inconsistent, leading to pose drifts or estimation errors. The proposed method can effectively calculate most of the hand–object 3D center locations in the dataset to crop the hand–object images. However, in some images, the manipulating hand and the object to be manipulated are not in contact, and the distance between them is large. For example, as shown in Figure 10, the manipulating hand (right hand) was far away from the object. Thus, the model failed to use the calculated hand–object 3D center for cropping in these images. It is impossible to acquire the cropped depth images containing the complete hand and object using the predicted hand–object 3D center for cropping when the hand and object are far away, without any contact. This decreases the accuracy of the follow-up pose estimation in these cases.

**Figure 11:**
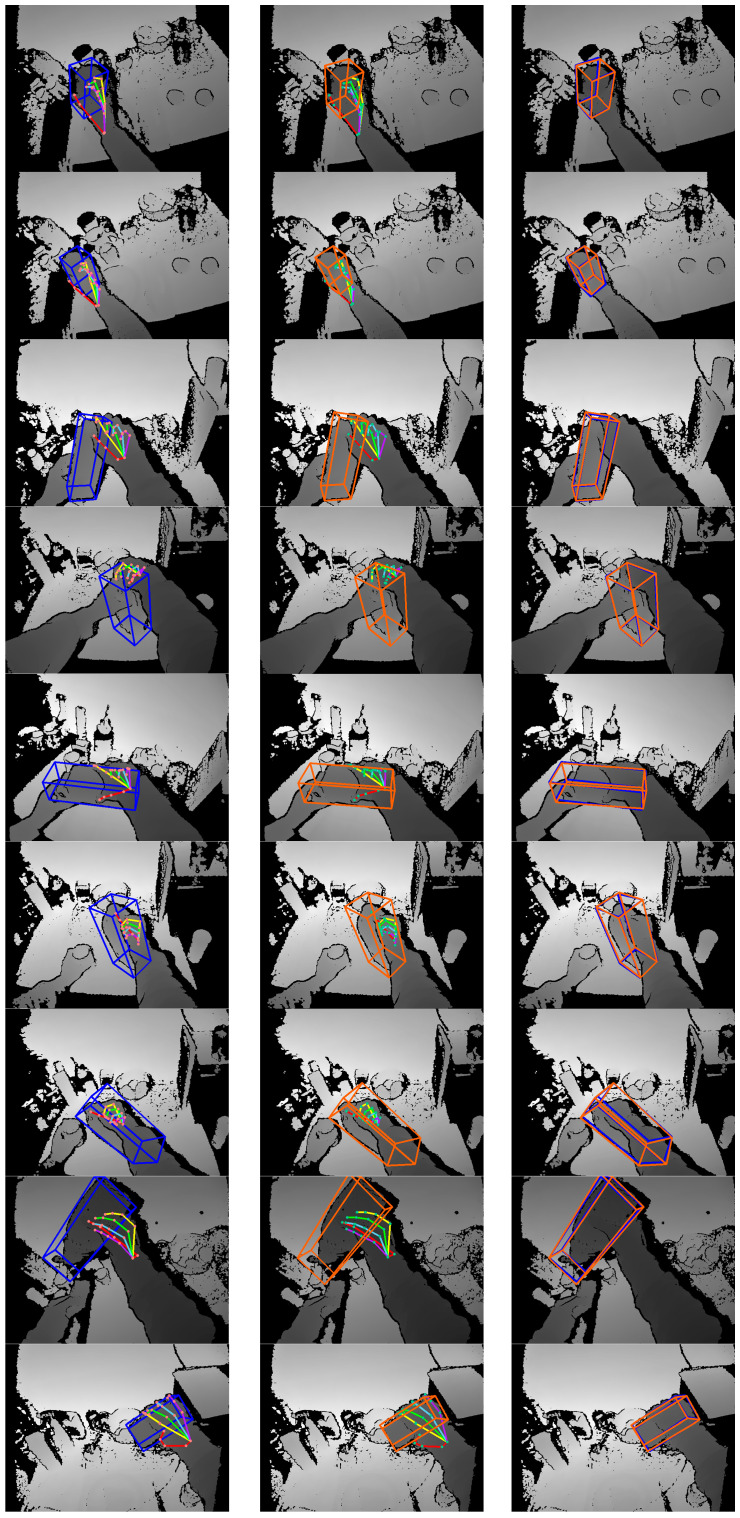
Some of the prediction results on the test set.

The proposed method focuses on pose estimation during the hand–object interaction state. The prediction results are shown in Figure 11. Column 1 shows the ground-truth pose labels for the hand and object. Column 2 shows the prediction results for the proposed method. Column 3 compares the predicted poses and ground-truth pose labels of the manipulated object. The proposed method can calculate the location of the hand–object 3D center and perform hand–object pose estimation from depth images. Given the hand–object 3D center, the hand–object pose estimation module based on anchor regression can perform pose estimation on an NVIDIA TITAN GPU at a speed of 31.40 FPS on average. The inference time of this module was about 31.85 ms for each testing depth image, including about 8.58 ms for image reading and preprocessing, and about 23.27 ms for the network forward propagation and the post-processing of anchor-regressed results.

## 5. Conclusions

In this study, a hand–object pose estimation method based on anchor regression is proposed. It can estimate the poses of a hand and its manipulated object expressed by the 3D locations of keypoints from egocentric depth images. First, to overcome the difficulties in acquiring hand–object centers in real scenes, a hand–object 3D center detection method based on YOLOv3 is proposed. With this method, the hand–object 3D center can be obtained, which can be used to effectively crop the hand–object local image from the original depth image. For the hand–object pose estimation task, a hand–object pose estimation method based on anchor regression is proposed. This method places anchor points uniformly on the depth image, assigns different weights to the anchor points, and finally integrates all the anchor point outputs through a weighted sum to produce accurate and robust pose estimation results. Anchor points in dense distribution can effectively extract local image features in different areas. Moreover, the learned weights allocated to the anchor points can reduce the influence of invalid anchor point outputs on the results, enabling accurate hand–object pose estimations. The experimental results demonstrated that the MKE values of hand and object pose estimations on the FPHA-HO dataset were 11.85 mm and 18.97 mm, respectively. Therefore, the proposed method can accurately estimate hand–object poses in an interaction state from egocentric depth images. This method has promising application prospects in the field of robot imitation learning, human–machine interaction, and augmented reality.

The method proposed in this paper performed well in most cases. However, its performance of 3D hand–object pose estimation was poor when the hands were severely occluded by large objects or there were missing areas in the depth image. In the future, temporal constraints can be introduced to enhance the robustness of the model in these scenarios. The depth offset parameter $D_{b}$ was empirically fixed at 60 mm in this paper based on experimental results on the FPHA-HO dataset. FPHA-HO is an egocentric dataset where hand–object interaction typically occurs within a limited, ergonomic range from the camera. Due to the relatively stable calibration parameters and subject distance of the egocentric depth camera, the model exhibits robustness to changes in $D_{b}$. In the future, an adaptive strategy for $D_{b}$ will be introduced to further enhance the generalization ability of the model. Although the proposed method acquired good hand–object pose estimation results based on local features, the use of the image’s global features was not completely explored. Future studies will explore the model’s ability to extract global features and seek a more optimal scheme to summarize anchor point outputs, so as to decrease the influence of large anchor point output errors on the final pose estimation accuracy. This study focuses on hand–object 3D pose estimation from single-frame egocentric depth images, without including action recognition. In the future, we will consider expanding the framework to integrate an action recognition module.

## Figures and Tables

**Table 1:** MKE values of different methods on the test set.

Method	Hand MKE (mm)	Object MKE (mm)
H+O (hand only) [22]	16.15	--
H+O (object only) [22]	--	28.27
H+O (base) [22]	16.87	25.54
H+O (interact) [22]	15.81	24.89
Ours (predicted center)	11.85	18.97
Ours (ideal center)	9.42	14.62

## Data Availability

The original contributions presented in this study are included in the article. Further inquiries can be directed to the corresponding author.

## Abbreviations

The following abbreviations are used in this manuscript:

CNN	Convolutional Neural Network
RNN	Recurrent Neural Network
YOLO	You Only Look Once
ICA	Iterative Cropping Algorithm
FCL	Fully Connected Layer
SRN	Stacked Regression Network
AWR	Adaptive Weighted Regression
SSD	Single Shot Multibox Detector
BB8	8 Corners of the Bounding Box
GCN	Graph Convolution Network
FPN	Feature Pyramid Network
HOAR	Hand–Object Pose Estimation Module Based on Anchor Regression
FPHA	First-Person Hand Action
MKE	Mean Keypoint Error
PCK	Percentage of Correct Keypoint Estimation
